# Acute Leptin Treatment Enhances Functional Recovery after Spinal Cord Injury

**DOI:** 10.1371/journal.pone.0035594

**Published:** 2012-04-20

**Authors:** Carmen María Fernández-Martos, Pau González, Francisco Javier Rodriguez

**Affiliations:** Molecular Neurology Laboratory, Hospital Nacional de Parapléjicos (HNP), Toledo, Spain; University of North Dakota, United States of America

## Abstract

**Background:**

Spinal cord injury is a major cause of long-term disability and has no current clinically accepted treatment. Leptin, an adipocyte-derived hormone, is best known as a regulator of food intake and energy expenditure. Interestingly, several studies have demonstrated that leptin has significant effects on proliferation and cell survival in different neuropathologies. Here, we sought to evaluate the role of leptin after spinal cord injury.

**Findings:**

Based on its proposed neuroprotective role, we have evaluated the effects of a single, acute intraparenchymal injection of leptin in a clinically relevant animal model of spinal cord injury. As determined by quantitative Real Time-PCR, endogenous leptin and the long isoform of the leptin receptor genes show time-dependent variations in their expression in the healthy and injured adult spinal cord. Immunohistochemical analysis of post-injury tissue showed the long isoform of the leptin receptor expression in oligodendrocytes and, to a lesser extent, in astrocytes, microglia/macrophages and neurons. Moreover, leptin administered after spinal cord injury increased the expression of neuroprotective genes, reduced caspase-3 activity and decreased the expression of pro-inflammatory molecules. In addition, histological analysis performed at the completion of the study showed that leptin treatment reduced microglial reactivity and increased caudal myelin preservation, but it did not modulate astroglial reactivity. Consequently, leptin improved the recovery of sensory and locomotor functioning.

**Conclusions:**

Our data suggest that leptin has a prominent neuroprotective and anti-inflammatory role in spinal cord damage and highlights leptin as a promising therapeutic agent.

## Introduction

Leptin, the product of the obese (ob) gene [1], is a polypeptide hormone primarily secreted by adipocytes that exerts its main biological functions in the brain [2], [3]. This 16-kDa non-glycosylated protein is first released into the blood and then transported into the brain via the blood-brain barrier to regulate food intake and energy balance [4]. Leptin acts by binding to its receptors that are structurally related to the cytokine receptor class I family. Alternative splicing of the diabetes (db) gene generates distinct isoforms of the leptin receptor, including long (ObRb) and short isoforms (ObRa and ObRc-f) that differ in the length of their intracellular cytoplasmic domains, a region that contains specific motifs involved in leptin signaling [1], [5]. The long isoform of the leptin receptor (ObRb) is thought to transmit the majority of leptin's biological signals. In the brain, the binding of leptin to the ObRb receptor activates janus-tyrosine kinase 2 (JAK2), which in turn phosphorylates the insulin receptor substrate-1 and -2 (IRS 1/2) that results in the activation of the phosphatidylinositol 3-kinase (PI3K)-akt pathway [6], [7]. JAK2 activation also leads to the phosphorylation of two tyrosine residues in the cytoplasmic tail of the ObRb receptor [8], producing the activation of the mitogen-activated protein kinase (MAPK)/extracellular signal-regulated kinase (ERK) [9], [10] and the signal transducer and activator of transcription 3 (STAT3) signaling pathways [8].

Indeed, several studies have demonstrated significant effects of leptin on reproduction [11], thermogenesis [12], synaptic plasticity [13] and neuroprotection [14]–[17]. In this regard, leptin promotes cell survival and proliferation in the nervous tissue via signaling pathways downstream of leptin receptors [14]–[17]. Leptin has been shown to exert neuroprotective effects in ischemia [16], Parkinson's and Alzheimer's diseases [18]–[24] and epilepsy [25], and systemic administration of leptin decreases infarct volume after focal cerebral ischemia in mice [16]. Taken together, these findings highlight the therapeutic potential of leptin as a treatment for a variety of central nervous system (CNS) disorders.

Spinal cord injury (SCI) is a major cause of long-term functional disability for which no clinically satisfactory treatment is yet available [26]. The loss of sensory-motor function after SCI, results from primary mechanical damage and the ensuing secondary neural cell death [27], [28]. Despite significant progress, the precise mechanisms underlying this secondary cell death remain unclear. In this regard, it is plausible that leptin may influence cell survival in the spinal cord after injury. Accordingly, the aim of this study was to determine the effects of an acute, intraparenchymal (IP) injection of leptin after SCI. In agreement with previous findings, we found that leptin administration enhances the expression of neuroprotective genes, reduces inflammation and significantly improves sensory and motor function after SCI.

## Results

### Leptin and ObRb are up-regulated after SCI

**Figure 1:**
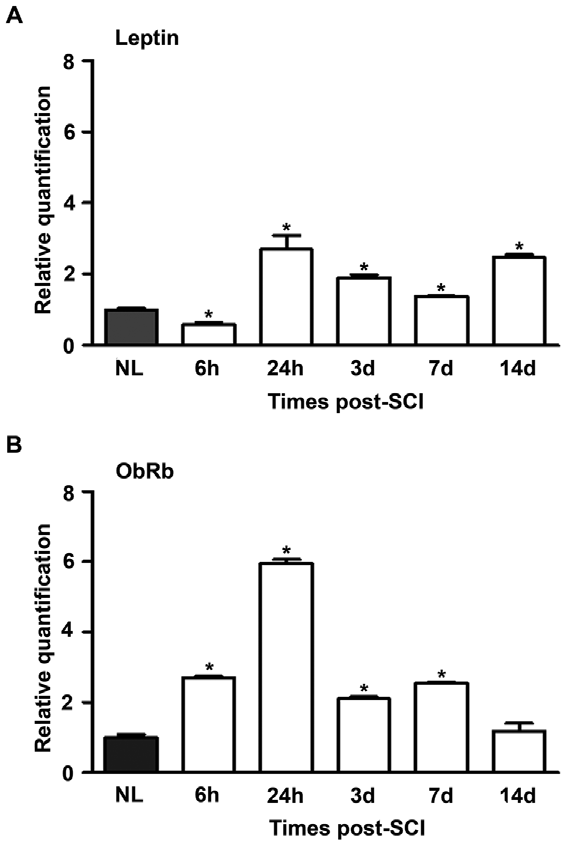
Leptin and ObRb are up-regulated after SCI. The expression of leptin (A) and ObRb (B) was quantified by RT-qPCR using specific primers (Table 1). The mRNA expression of ObRb was significantly increased 6 h post-SCI, while the mRNA expression of leptin was decreased. At 24 h, 3 d and 7 d post-SCI, the expression of both mRNAs was significantly increased. At 14 d post-SCI, leptin mRNA expression remained up-regulated, while ObRb mRNA expression had returned to the basal levels observed in the non-lesioned group (NL). Differences were calculated by setting the expression values of the NL samples at 1 and normalising against ribosomal 18S rRNA. The differences are shown as the mean ± SEM; *p<0.05 versus NL.

We confirmed that endogenous leptin and ObRb genes were expressed in non-lesioned spinal cord samples (NL). Strikingly, we detected marked differences in the expression of both leptin and OBRb mRNA following SCI when compared with the NL samples. More specifically, there was a down-regulation of leptin (Figure 1A) and an up-regulation of ObRb (Figure 1B) mRNA expression 6 hours (h) post-SCI, followed by a significant increase in the expression of both mRNA transcripts from 24 h to 7 days (d) post-SCI. However, at 14 d post-SCI, the ObRb mRNA expression returned to the basal level observed in the NL spinal cords (Figure 1B).

### ObRb is expressed in neural cells

**Figure 2:**
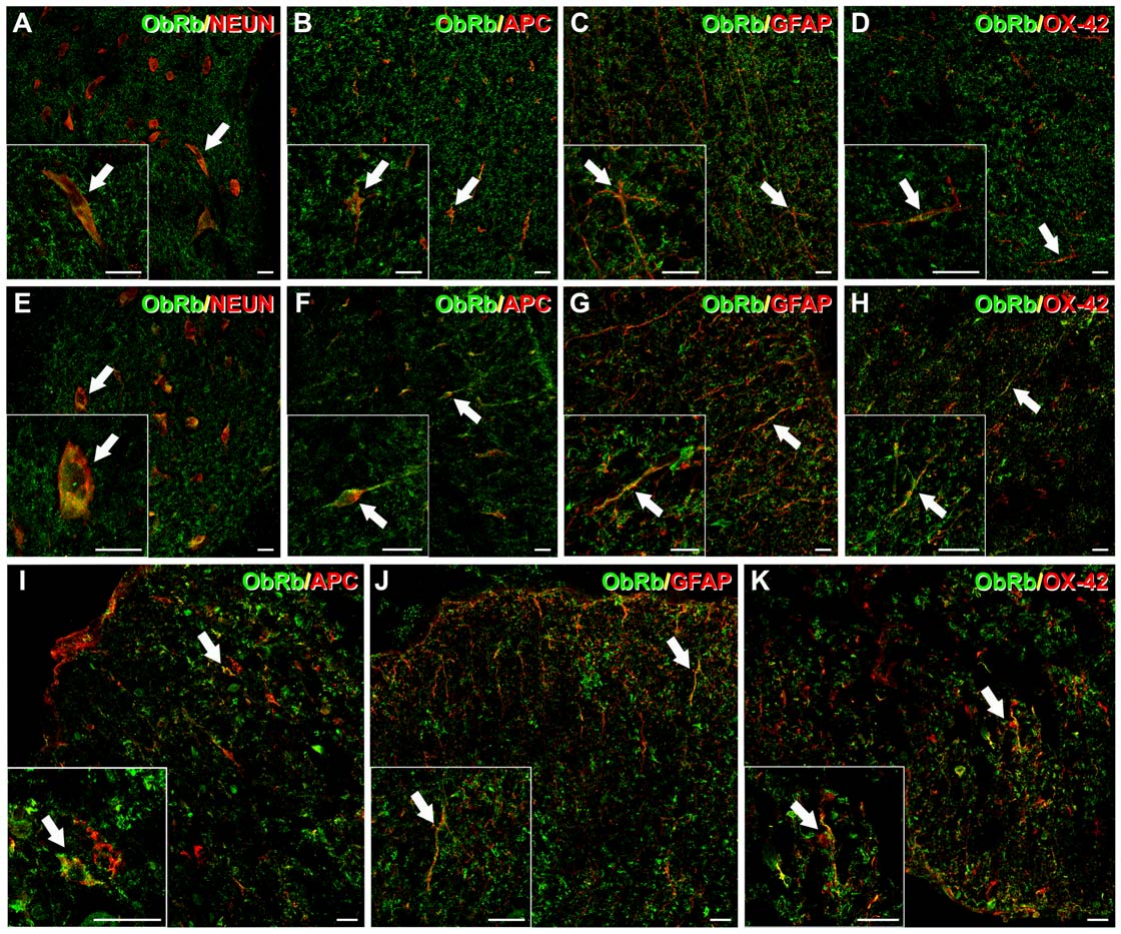
Immunohistochemical analysis of ObRb expression after SCI. Dual immunohistochemistry was performed in uninjured spinal cord sections, and 24 h post-SCI on spinal cord sections from vehicle-treated and leptin-treated animals. ObRb immunolabelling was observed in uninjured spinal cord sections (A to D) and in both rostral and caudal unaffected areas (E to H) and at the lesion epicentre (I to K). Cells expressing ObRb were identified as NeuN^+^ neurons (A and E), APC^+^ oligodendrocytes (B, F and I), GFAP^+^ astrocytes (C, G and J) and OX-42^+^ microglia/macrophages (D, H and K). Scale Bars = 20 µm.

Dual immunohistochemistry (IHC) analyses were performed in spinal cord sections from uninjured and injured vehicle/leptin-treated rats at 24 h post-SCI. In uninjured spinal cord sections ObRb was found primarily in neurons and oligodendrocytes and, to a lesser extent, in astrocytes and microglia/macrophages (Figure 2A–D). Otherwise, at 24 h post-SCI ObRb was found primarily in oligodendrocytes and, to a lesser extent, in astrocytes and microglia/macrophages in both rostral and caudal unaffected areas (Figure 2F–H) and in the lesion epicentre (Figure 2I–K). Finally, neuronal ObRb receptor expression was detected in both rostral and caudal unaffected areas (Figure 2E). Notably, the cellular ObRb expression pattern was identical between treatment groups (data not shown).

### Leptin increases the expression of neuroprotective genes after SCI

**Figure 3:**
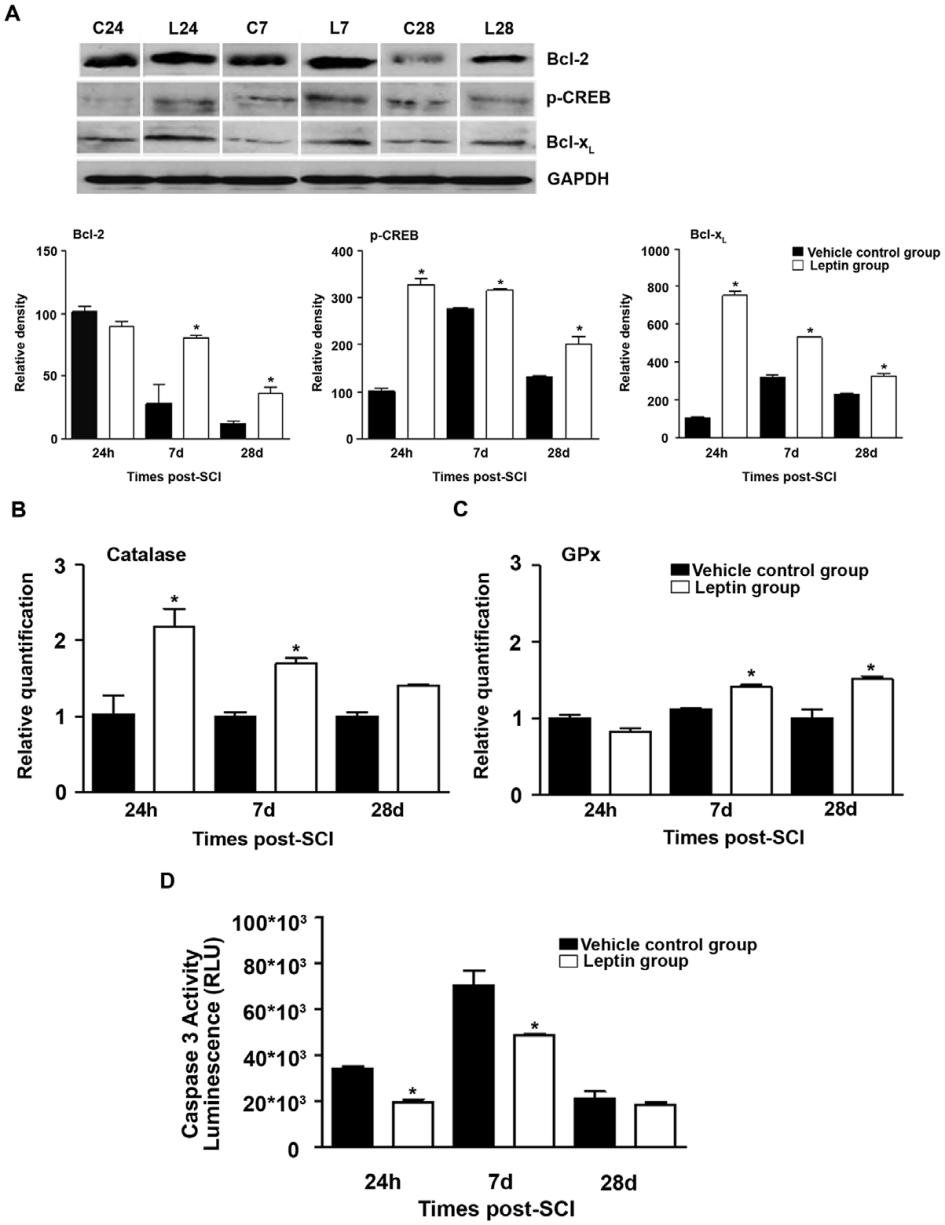
Leptin administration increased the expression of neuroprotective genes. (A) Leptin treatment induced an increase in Bcl-2, Bcl-x_L_ and p-CREB proteins when compared with vehicle-treated controls. Bcl-2 protein levels were increased significantly beginning at 7 d post-SCI and remained elevated until 28 d post-SCI. Bcl-x_L_ and p-CREB protein levels were increased significantly beginning at 24 h post-SCI and remained elevated until 28 d post-SCI when compared with vehicle-treated controls. (B and C) An increase in antioxidant enzyme mRNA expression was observed in leptin-treated animals when compared with vehicle treated controls. In leptin-treated animals, catalase gene expression (B) was significantly increased after 24 h and remained elevated until 28 d post-SCI. GPx gene expression (C) was significantly increased after 7 d and remained elevated until 28 d after injury. (D) In the early stages after injury (24 h and 7 d post-SCI), leptin treatment decreased caspase-3 activity, while at later stages (28 d post-SCI) no differences were detected when compared with vehicle-treated controls. The white lines in the western blot analysis (A) represent the lanes that were run on the same gel but were non-contiguous. The data in (A) are presented as a percentage of the corresponding value in the vehicle-treated control group 24 h post-SCI. The values in A–D represent the mean ± SEM; *p<0.05 versus the vehicle control group.

Western blot analysis of neuroprotective gene expression showed that leptin-treated animals displayed a significant increase in the protein levels of B-cell lymphoma/leukemia 2 (Bcl-2) at 7 and 28 d post-SCI and in the protein levels of B-cell lymphoma-extra-large (Bcl-x_L_) and active phosphorylated CREB (p-CREB) at all analysed time points (Figure 3A). Quantitative Real Time-PCR (RT-qPCR) analysis revealed a significant up-regulation of catalase and glutathione peroxidase (GPx) mRNA expression in leptin-treated animals (Figure 3B and 3C). In addition, there was a significant decrease in caspase-3 activity in leptin-treated rats at 24 h and 7 d after injury when compared with control rats (Figure 3D).

### Leptin ameliorates the expression of inflammatory genes

**Figure 4:**
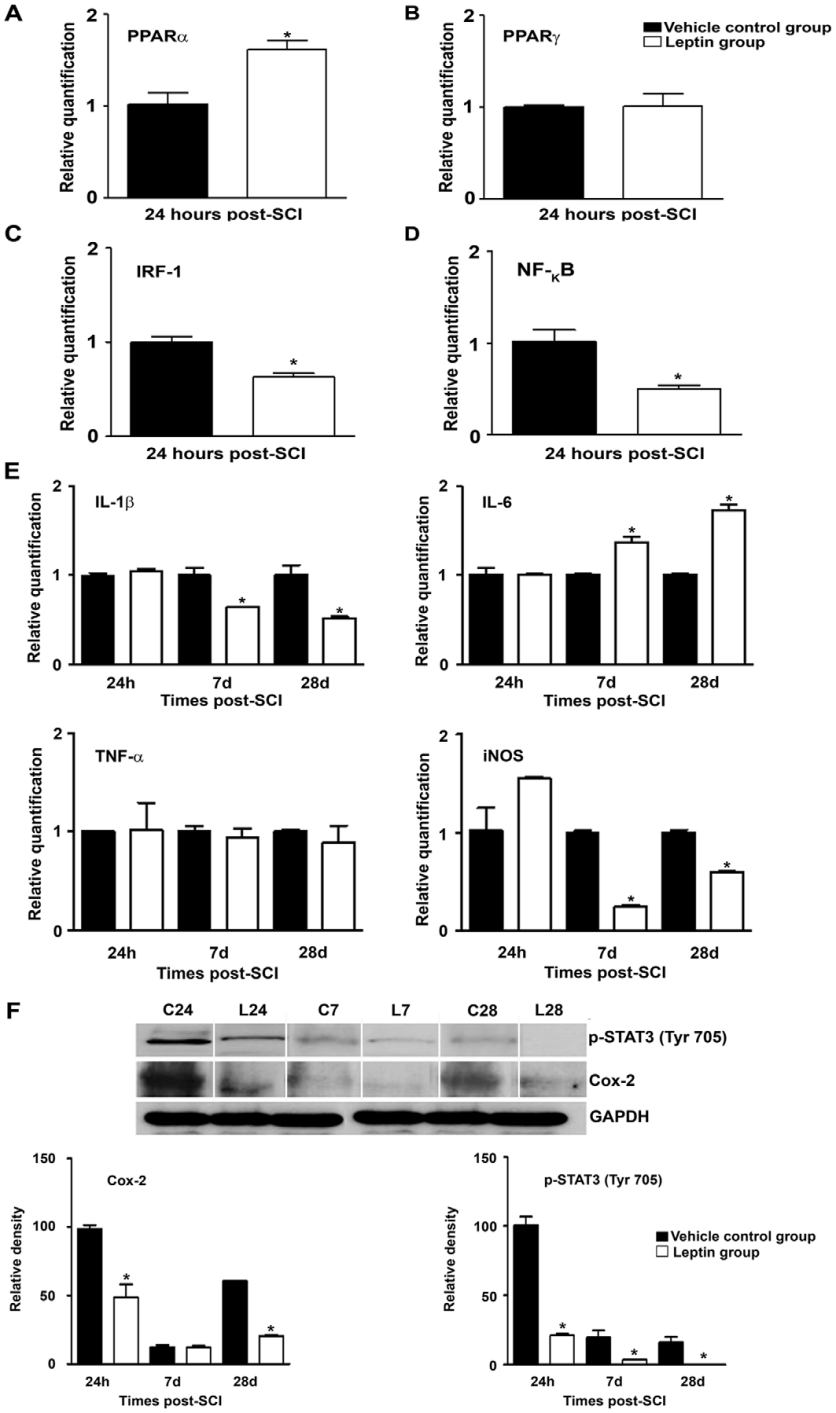
Leptin prevents the induction of the expression of inflammatory genes. Leptin administration significantly increased PPARα mRNA expression (A), while no differences in PPARγ mRNA expression (B) were detected 24 h post-SCI when compared with the vehicle-treated controls. Moreover, leptin treatment significantly decreased IRF-1 (C) and NF-_k_β (D) mRNA expression 24 h post-SCI. The gene expression of IL-1β and iNOS (E) was significantly decreased 7 d post-SCI and remained down-regulated until 28 d post-SCI. However, IL-6 mRNA expression was up-regulated after leptin administration and no effect on TNF-α mRNA levels (E) was detected when compared with vehicle-treated controls. (F) Leptin administration induced a significant decrease in Cox-2 and p-STAT3 (Tyr 705) protein levels when compared with vehicle-treated controls. Cox-2 and p-STAT3 (Tyr 705) proteins were significantly decreased 24 h post-SCI and remained down-regulated until 28 d post-SCI. The white lines in the western blot analysis (F) represent the lanes that were run on the same gel but were non-contiguous. The data in (F) are presented as a percentage of the corresponding value in the vehicle-treated control group 24 h post-SCI. The values in A–F represent the mean ± SEM; *p<0.05 versus the vehicle control group.

We evaluated the effect of leptin treatment on the expression of a wide range of pivotal inflammatory-related molecules, such as peroxisomal proliferator activated receptors (PPARs), pro-inflammatory transcription factors, cytokines and enzymes using western blot or RT-qPCR techniques. Our results showed a significant increase in the expression of PPARα mRNA (Figure 4A) 24 h post-SCI but no differences in PPARγ mRNA expression (Figure 4B). At this time point, we also observed a significant decrease in the expression of interferon regulatory factor-1 (IRF-1) and nuclear factor kappa-light-chain-enhancer of activated B cells (NF-_k_β) in leptin-treated animals when compared with the controls (Figure 4C and 4D) however, no differences in inducible nitric oxide synthase (iNOS), interleukin (IL)-1β and IL-6 mRNA expression were detected (Figure 4E). At 7 and 28 d post-SCI, there was a significant decrease in iNOS and IL-1β mRNA expression coupled with an increase in IL-6 expression in leptin-treated animals when compared with control animals (Figure 4E). No changes in tumor necrosis factor-alpha (TNF-α) mRNA expression were observed at any of the time points analysed (Figure 4E). Lastly, these changes at the mRNA level were concomitant with a decrease in cyclooxigenase-2 (Cox-2) and active phosphorylated STAT3 (p-STAT3) protein levels (Figure 4F).

### Leptin enhances functional motor recovery and prevents the development of neuropathic pain after SCI

**Figure 5:**
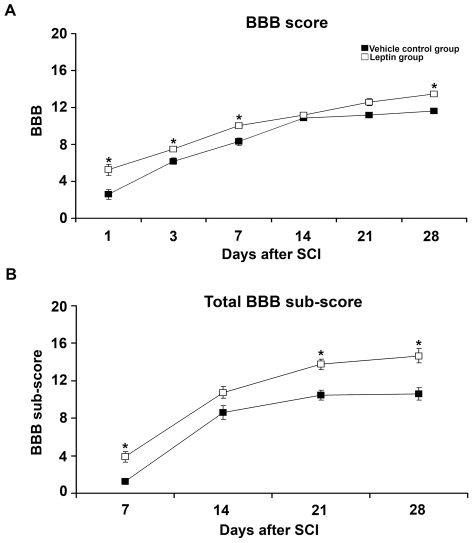
The effect of leptin administration on motor function recovery in the open-field test. (A) Recovery of motor functioning was determined using the Basso-Beattie-Bresnahan (BBB) locomotor scale. Leptin administration enhanced the recovery of motor functions at 1, 3, 7 and 28 d after SCI when compared with vehicle-treated controls. (B) The BBB sub-scores (7–28 d post-SCI) were significantly higher following leptin administration. In all cases, the values represent the mean ± SEM; *p<0.05 versus the vehicle control group.

**Figure 6:**
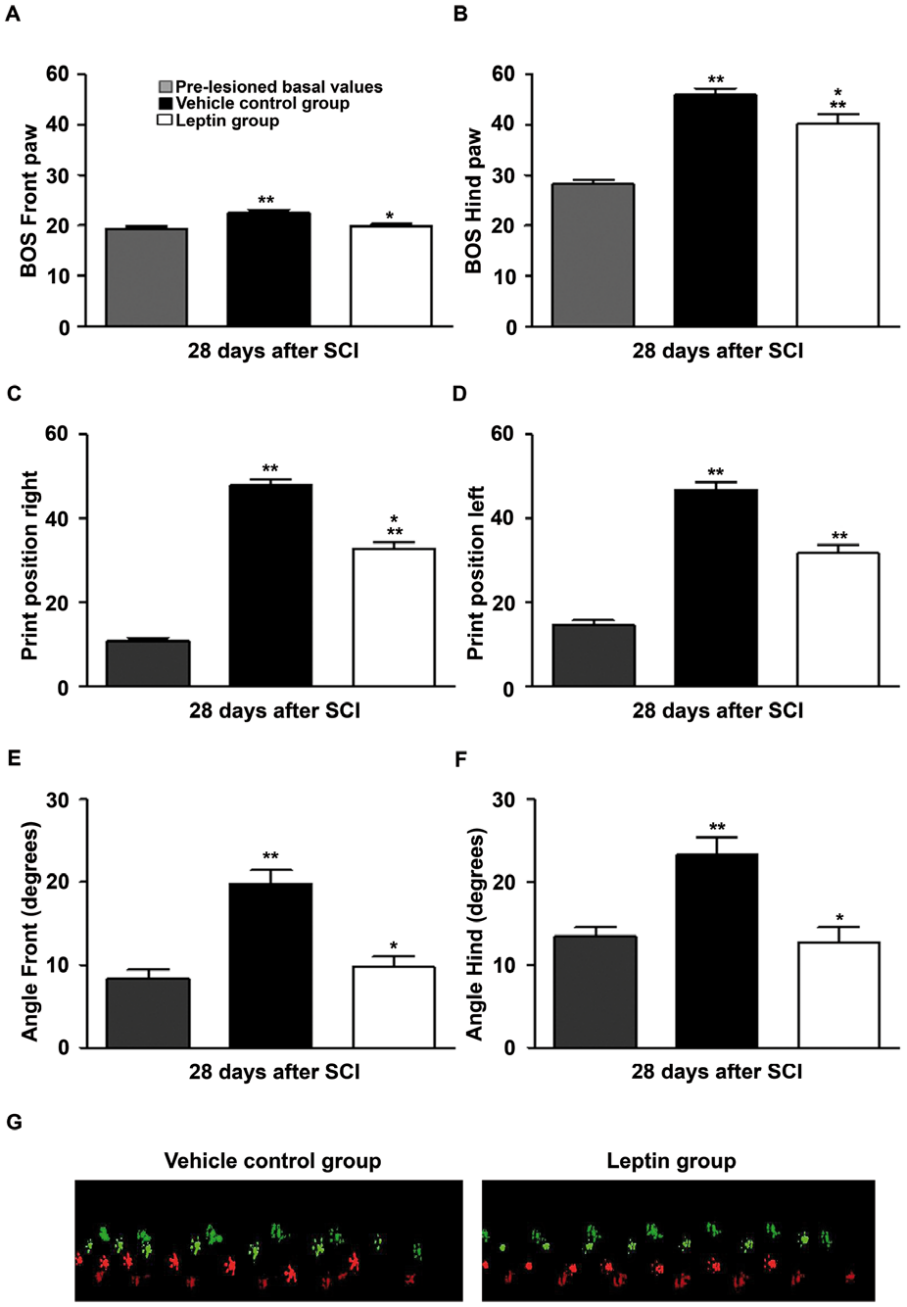
The effect of leptin administration on motor function recovery in the CatWalk system. Gait assessment was performed using the CatWalk® gait analysis system (version 7.1; Noldus) at 28 d post-SCI in 5 uninterrupted and consistent pace trials per animal. Leptin administration decreased the base of support (BOS) of both of the fore (A) and hind (B) paws when compared with vehicle-treated controls. The right/left print position (C and D) and the front/hind paw angle (E and F) in leptin-treated animals were significantly lower than in vehicle-treated controls. (G) Examples of the step sequence pattern analysis in representative vehicle-treated and leptin-treated animals. The values in A–F represent the mean ± SEM; **p<0.0001 versus the basal values in pre-lesioned conditions; *p<0.001 versus the vehicle control group.

**Figure 7:**
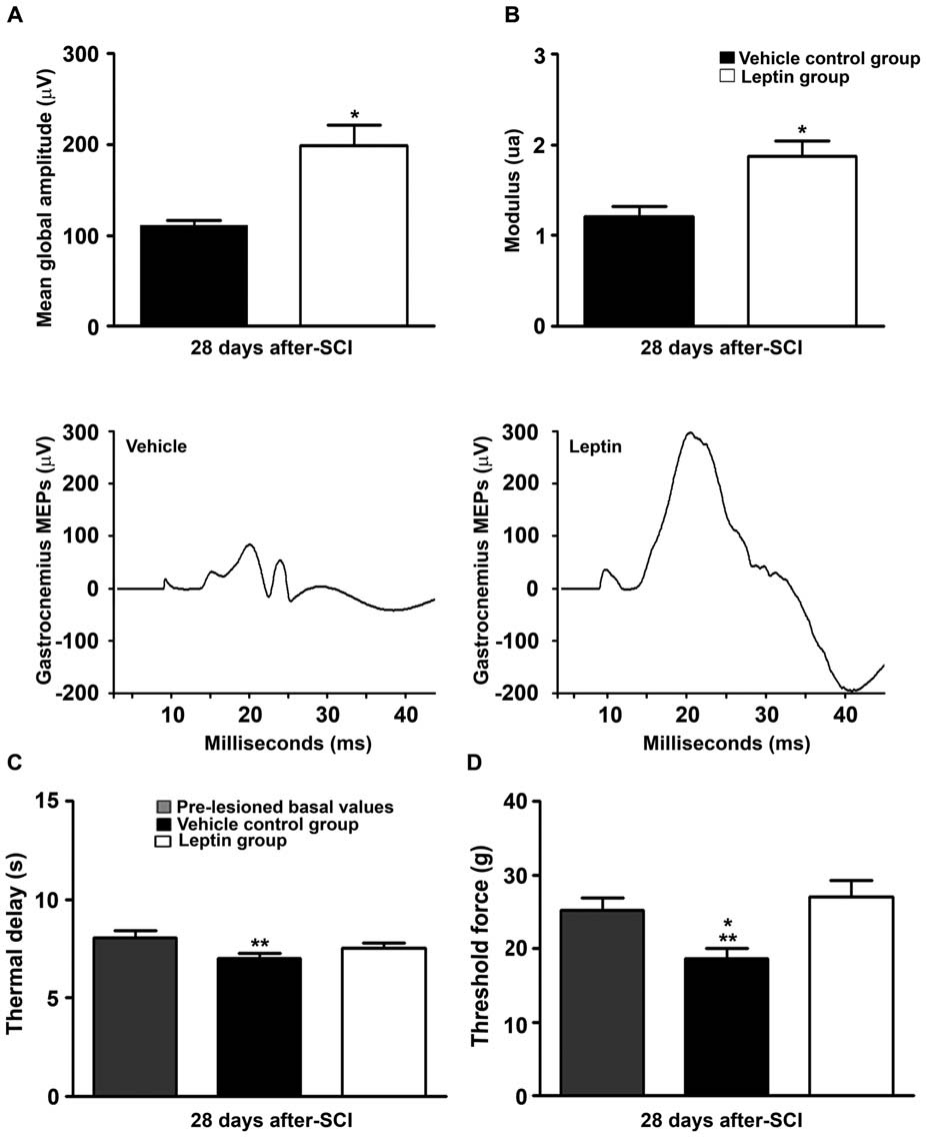
Electrophysiological assessment and neuropathic pain. Electrophysiological tests were carried out at 28 d post-SCI to analyse the motor evoked potentials (MEPs). Leptin administration significantly increased the average maximum amplitude (A) and rectified area under the curve (B) when compared with vehicle-treated controls. (C and D) At 28 d post-SCI, vehicle-treated controls exhibited a significant decrease in paw withdrawal latency when compared with the basal values determined in pre-lesioned conditions, indicative of thermal hyperalgesia. Leptin administration had no effect on the thermal delay when compared with the basal values determined in pre-lesioned conditions, indicating an attenuation of neuropathic pain. In terms of nociception, we observed similar mean latencies of heat-induced paw withdrawal in leptin-treated animals when compared with pre-lesioned conditions (8.01±0.36 seconds), while a significant decrease in latency was observed in the vehicle control group (6.84±0.25 seconds). Similarly, mechanical stimulation with Von-Frey filaments required a significantly lower mean threshold force to induce hind paw retraction in the vehicle control group (18±2.1 grams) following SCI when compared with the basal values determined in pre-lesioned conditions (25±1.67 grams); however, there were no such differences in leptin-treated animals (D). The values in A–D represent the mean ± SEM. A and B; *p<0.0025 versus the vehicle-treated controls. C and D; **p<0.05 versus the basal values in pre-lesioned conditions; *p<0.05 versus the vehicle-treated controls.

Prior to injury, all rats showed a maximum Basso, Beattie, Bresnahan (BBB) [29] score of 21, indicating normal motor function. At 1 d post-SCI the BBB score was less than 3 in all of the vehicle-treated rats, indicating an almost complete loss of motor function. Strikingly, all rats from the leptin-treated group attained BBB scores greater than 3, indicating a significant preservation of motor function, even at this early post-injury stage (Figure 5A). The initial improvement in motor function was sustained, with significantly higher scores in the leptin-treated group when compared with controls, except at 14 and 21 d after injury. These changes could be due to the intrinsic limitations of the BBB scale, as evidenced by the significantly higher total BBB sub-score of the leptin-treated group when compared with the vehicle group at 7, 21 and 28 d post-SCI (Figure 5B). In fact, when the total sub-scores were examined individually, only the leptin-treated animals could step 7 d after injury, with the coordination sub-scores reaching significance at 21 and 28 d post-SCI. Indeed, at these time points both toe clearance and paw positions were improved by leptin treatment (Figure S1A–D). Finally, leptin-treated rats were able to lift their tail during locomotion earlier than the vehicle-treated rats, suggesting better overall stability (data not shown). Thus, leptin-treated rats stepped earlier and displayed a more normal stepping pattern when compared with the vehicle-treated animals. Accordingly, the CatWalk gait assessment at 28 d post-SCI revealed a significant functional improvement in the leptin-treated group as shown by a decrease in the base of support (BOS), a reduction in the distance between consecutive fore and hind paw placement and a more parallel paw position relative to the advancing axis (Figure 6A–G). Indeed, electrophysiological recordings of hind limb motor evoked potentials (MEPs) revealed an improved capacity for transmission of neural motor impulses through the injury site in leptin-treated animals when compared with the vehicle-treated animals (Figure 7A and 7B). We also assessed the effects of leptin administration on hyperalgesia and allodynia in a random subset of animals at the completion of the study. Interestingly, leptin treatment prevented the development of the thermal hyperalgesia (Figure 7C) and allodynia (Figure 7D) that was observed in animals treated with the vehicle.

### Leptin decreases chronic microglia/macrophage activation and caudal white matter loss after spinal cord damage

**Figure 8:**
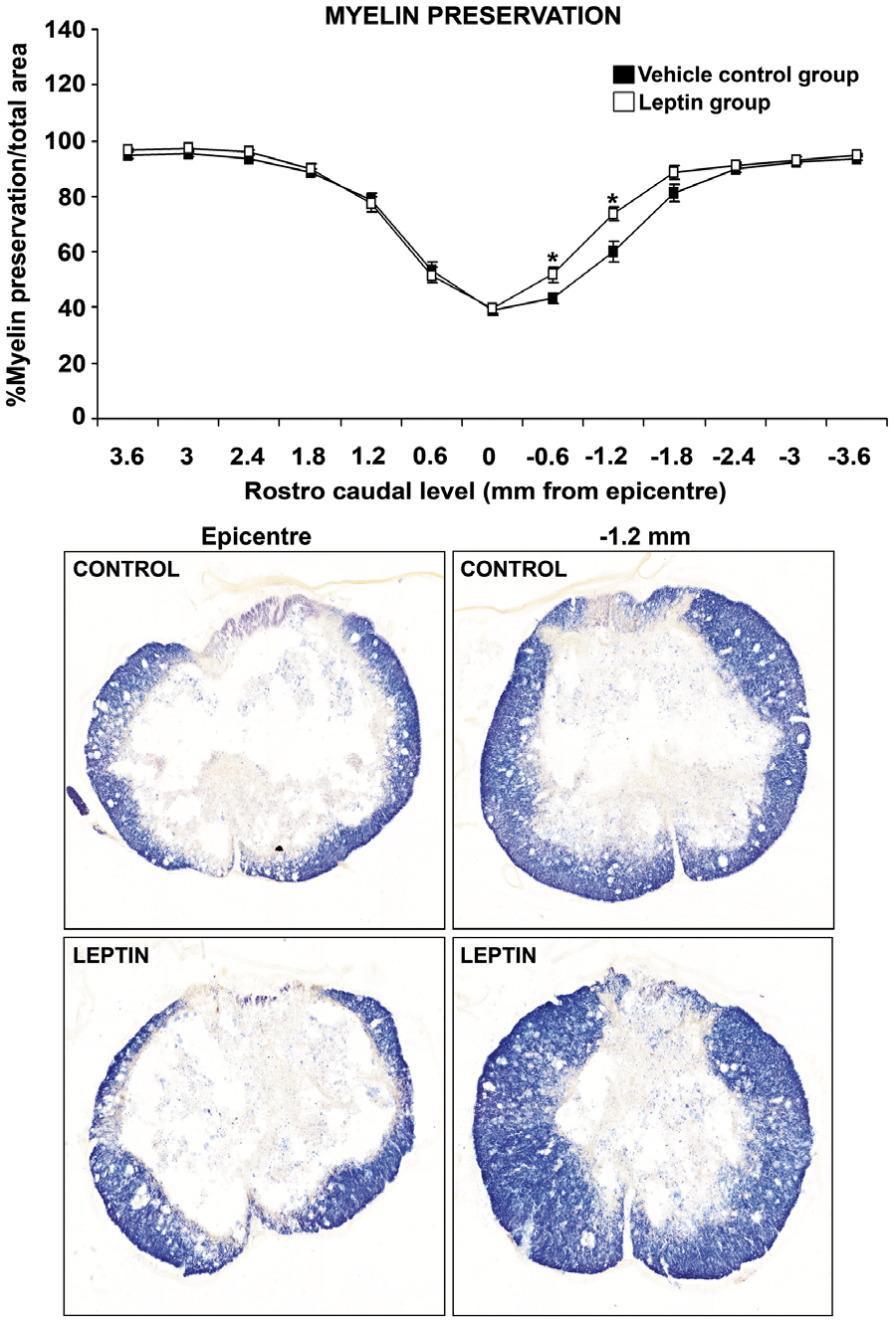
Leptin administration increases the preservation of caudal white matter. Leptin administration significantly increased the preservation of white matter when compared with vehicle treated controls, specifically in areas corresponding to caudal levels adjacent to the lesion epicentre. The values represent the mean ± SEM; *p<0.05 versus the vehicle-treated controls.

**Figure 9:**
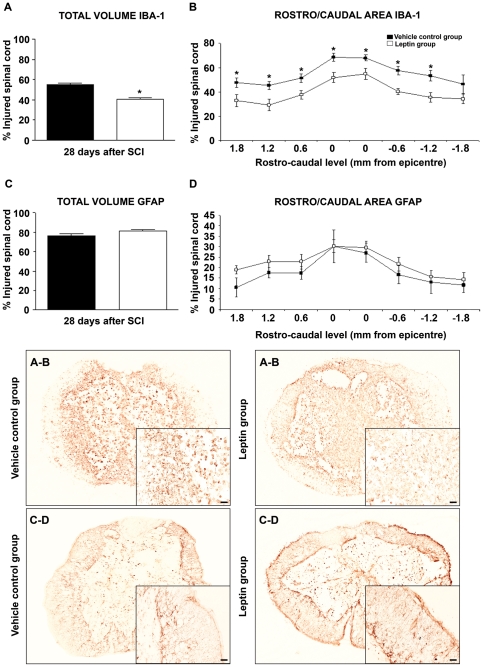
Leptin administration decreases microglial/macrophage activation but has no effect on astroglial reactivity. Leptin administration significantly diminished the presence of Iba1^+^ microglia/macrophages when compared with vehicle treated controls, as measured by the total volume occupied by Iba1^+^ microglia/macrophages (A) and the area occupied at multiple rostro-caudal levels (B). No differences between groups were observed in the total volume occupied by GFAP^+^ astrocytes (C) or the area occupied at each rostro-caudal level (D). The values represent the mean ± SEM; *p<0.05 versus the vehicle-treated control group. Scale bars = 50 µm.

Analysis of white-matter preservation at 28 d post-SCI revealed a higher percentage of myelin preservation in caudal levels adjacent to the lesion epicentre in leptin-treated animals (Figure 8). We also evaluated astroglial and microglial reactivity (Figure 9) at the same time point. Notably, leptin treatment induced a significant decrease in the overall total volume occupied by Iba1^+^ reactive microglia/macrophages (Figure 9A) and at multiple rostro-caudal levels of the injury site (Figure 9B). However, no differences were detected between vehicle and leptin-treated groups either in total volume (Figure 9C) or in the area occupied by GFAP+ astrocytes at each of the rostro-caudal levels analysed (Figure 9D).

**Figure 10:**
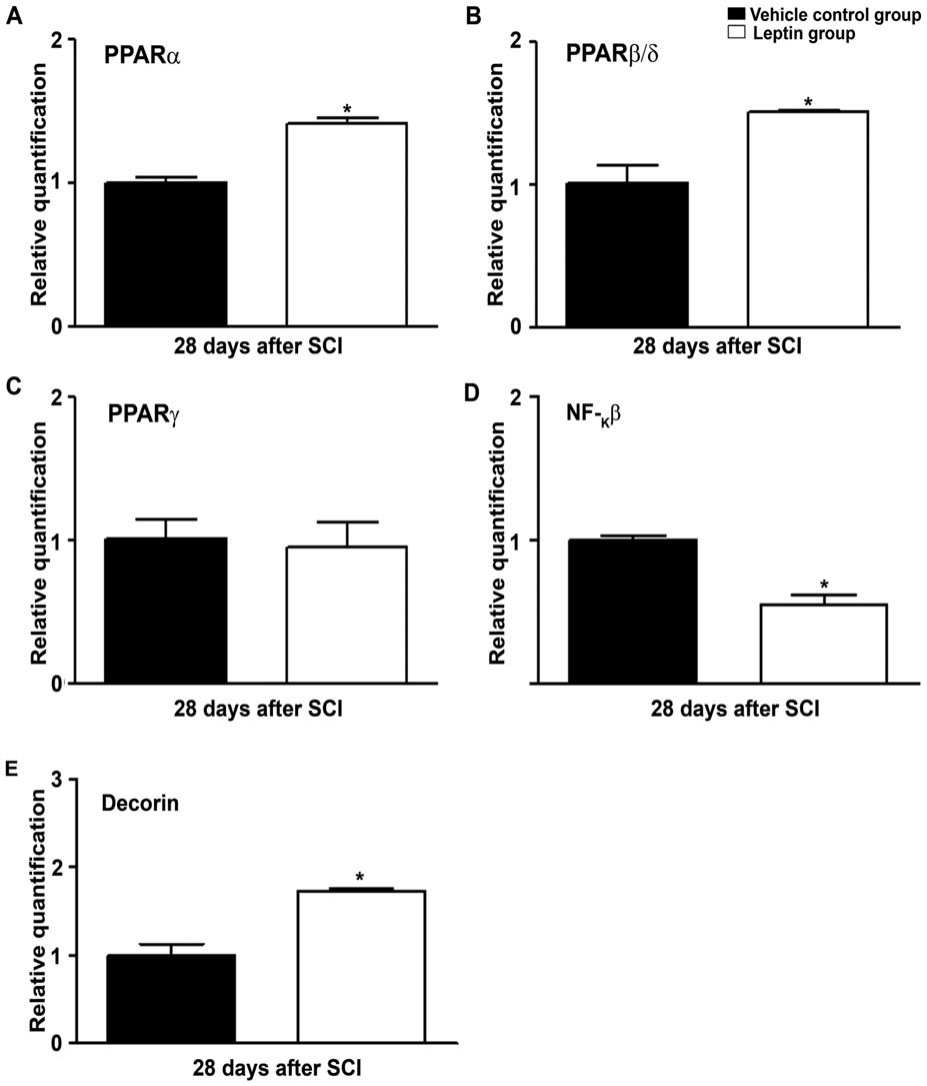
The effect of leptin treatment on PPARs, NF-kβ and decorin in the chronic phase of SCI. The expression of PPARα (A), PPARβ/δ (B), PPARγ (C), NF-_k_β (D) and decorin (E) were quantified by RT-qPCR 28 d post-SCI. As shown, a significant up-regulation of PPARα and PPARβ/δ mRNA expression was observed in leptin-treated animals. No differences were detected in the mRNA expression of PPARγ when compared with vehicle-treated controls. Moreover, a significant decrease in NF-_k_β mRNA expression and an increase in decorin mRNA expression were observed in leptin-treated animals. The values in A–E represent the mean ± SEM; *p<0.05 versus the vehicle-treated controls.

RT-qPCR analysis at 28 d post-SCI revealed a significant up-regulation of PPARα and PPARβ/δ mRNA expression in response to leptin treatment (Figure 10A and 10B), while no differences were detected in PPARγ mRNA expression (Figure 10C). Moreover, we observed a significant down-regulation of NF-_k_β mRNA (Figure 10D) and an increase in decorin mRNA expression (Figure 10E) in leptin-treated animals.

## Discussion

Our results demonstrate that endogenous leptin and ObRb receptor is expressed in NL spinal cords and that there is a time-dependent up-regulation of these genes following a moderate contusive SCI. At 6 h post-SCI, we observed a significant decrease in leptin mRNA paralleled by an increase in ObRb mRNA expression. Thus, we decided to administer a single injection of recombinant leptin 5 min after SCI. This resulted in an activation of intracellular signaling pathways that promoted the expression of neuroprotective genes and antioxidant enzymes, as well as, prevented the expression of pro-inflammatory transcription factors, cytokines and enzymes. We also demonstrated a significant improvement in sensory and motor functional outcomes with leptin treatment after SCI.

In spinal cord neuropathology, sensory-motor functional loss results from the primary mechanical impact and the multifaceted secondary degenerative response that is promoted by a combination of deleterious processes, including inflammation, excitotoxicity and the generation of a glial scar that strongly inhibits axon growth [30]. The secondary degenerative response, due to apoptosis and an early inflammatory response that occurs between 8 h and 1 week post-SCI, is characterised by demyelination, axonal injury and the destruction of nervous tissue that survived the initial impact [31]. Based on the recently reported anti-apoptotic activity of leptin in different cell types [14], [15], [17], we evaluated ObRb immunoreactivity after SCI. We observed ObRb immunoreactivity in oligodendrocytes and, to a lesser extent, in astrocytes, microglia/macrophages and neurons. Moreover, we quantified the mRNA expression of antioxidant enzymes that mediate the metabolism of free radicals [28] and the level of anti-apoptotic proteins because many survival genes are down-regulated in the acute phase of SCI and the onset of secondary cell death [31]. Interestingly, an IP injection of leptin induced the mRNA expression of the antioxidant enzymes catalase and GPx [28], the anti-apoptotic proteins Bcl-2 and Bcl-x_L_ and increased p-CREB protein levels, a potent pro-survival transcription factor downstream of the ERK 1/2 target gene [15]. In addition, leptin treatment decreased cell death, as evidenced by a decrease in caspase-3 activity, a significant preservation of the white matter in the caudal levels adjacent to the lesion epicentre and an increase in PPARβ/δ mRNA expression, a transcription factor expressed by oligodendrocytes and glial precursors known to promote neuroprotection [32] and myelination [33]. Therefore, we cannot rule out that the survival effects of leptin could be largely due to specific activation of the ObRb receptor in neurons and glial cells.

Based on these observations and to gain further insight into the mechanism of action of leptin, we evaluated the inflammatory response, a key component of SCI pathogenesis and secondary cell death [34]. Strikingly, leptin treatment induced a significant decrease in the volume occupied by Iba1^+^ microglia/macrophages and a significant increase in mRNA levels of PPARα, a key modulator of inflammation, by inhibiting signaling via NF-_k_β [35]. Accordingly, leptin decreased the expression of NF-_k_β and IRF-1 mRNA and the downstream inflammatory genes, iNOS and Cox-2, at both early and chronic stages of SCI. Leptin also promoted a considerable increase in IL-6 but decreased IL-1β mRNA expression beginning at 7 d post-SCI. Together, our results point to a prominent inflammatory modulation by leptin that could be responsible for at least part of the leptin-induced expression of pro-survival genes.

Another major SCI impairment is the formation of the glial scar, a key obstacle that acts as a barrier to regenerating axons and impairs functional recovery after SCI [36]. Our results show that ObRb is expressed in astrocytes after SCI and that leptin treatment promotes a significant reduction in microglia/macrophage reactivity with a concomitant decrease in NF-_k_β mRNA expression, a well-known transcriptional regulator of molecules responsible for proteoglycan synthesis [37]. Although leptin does not seem to have any effect on GFAP immunoreactivity, it does increase decorin mRNA levels [38], [39]. Decorin is a small desmatan/chondroitin sulfate proteoglycan that acts as a natural inhibitor of scar formation by reducing the expression of neurocan and phosphacan proteoglycans [38]–[40]. Moreover, leptin also induced the up-regulation of IL-6, p-CREB and Bcl-2 from 7 d post-SCI. These proteins have been linked to synaptic plasticity, dendritic growth, neuritogenesis and axon regeneration [41], [42]. Indeed, several studies have described the modulation of axonal growth and synaptic plasticity by leptin [43]–[45].

Overall, as a result of its pro-survival and immunomodulatory action, the remarkable finding of this work is that a single acute leptin treatment can promote a significant recovery of both motor and sensory function after SCI. Importantly, in contrast to earlier reports on a sciatic chronic constriction injury model [46], [47], leptin administration prevented the development of thermal hyperalgesia and mechanical allodynia in our model of SCI. In this regard, these studies showed that the effect of leptin on spinal NMDA currents was mediated through leptin receptors, concretely by activating the JAK2/STAT3 signaling pathway. In line, leptin induced the up-regulation of the expression of NR1 and p-STAT3, which was also mediated through NMDAR. Although therapeutic approaches to prevent chronic pain after injury are limited, at the molecular level, the selective inhibition of Cox-2, iNOS and p-STAT3 activity can attenuate neuropathic pain [48], [49]. Accordingly, we observed a decrease in Cox-2 protein levels and iNOS mRNA expression with a concomitant attenuation of p-STAT3 in leptin-treated animals. Given that contusion injury is a valid model for the analysis of neuropathic pain [50], the present findings provide new evidence that leptin has beneficial effects in pain management after moderate contusive SCI. In this context, it should be noticed that the different experimental models might account for the divergence observed in the effects of leptin and, thus, further investigation will be necessary to clarify this issue.

In conclusion, this study shows that acute leptin administration enhances the expression of neuroprotective genes, reduces inflammation and significantly improves sensory and motor functional outcomes after SCI. Thus, our observations suggest that leptin might be a promising therapeutic agent to treat CNS injury given its clinical use in humans [51], [52], the ease with which it can be delivered systemically and the well-known therapeutic doses [53]–[55] and its ability to cross the blood brain barrier [56].

## Materials and Methods

### Experimental design and spinal cord injury

Animals were distributed into two experimental blocks. In the first experimental block, the expression of endogenous leptin and ObRb genes was studied at different times after SCI by RT-qPCR of total mRNA extracted from 21 animals (7 groups: non-lesioned group (NL), 6 and 24 hours (h) and 3, 7 and 14 days (d) after SCI; n = 3 per group). In the second experimental block, 62 animals were used to evaluate the effects of an acute IP administration of leptin after SCI in two independent experiments (see Tables S1 and S2).

All experiments were performed with adult male Wistar rats (300–350 g) in accordance with institutional guidelines for the care and use of experimental animals (Permit numbers 51/2009 and 45/2008). A moderate spinal cord contusion was created using an Infinite Horizon Impactor (Precision Systems and Instrumentation LLC), as previously described [57].

### Drug administration

Recombinant rat leptin (Sigma) dissolved in 15 mM Tris HCL (pH 8.0) or the vehicle alone (15 mM Tris HCL pH 8.0), was injected 5 min after SCI. These treatments were delivered stereotaxically (David Kopf Instruments) at the dorsal midline to a depth of 0.5 mm. They were administered in 3 separate injections (2 µl/4 µg/each): 1 to the wound epicentre, 1 mm rostral to the injury epicentre and 1 mm caudal to the injury epicentre. Injections were performed using a Nanofil® bevelled needle (135 Outer and 55 Inner micrometre diameters) attached to a 10 µl syringe (World Precision Instruments) and a microinjector (KD Scientific).

### Evaluation of sensory-motor recovery

Animals were tested in multiple sensory-motor tasks, including the BBB open field test, the CatWalk® gait analysis system, the Hargreaves heat and von Frey mechanical elicited withdrawal response.

#### 1. Locomotor function assessment

In the open-field test, the 21-point BBB scale [29] was used to measure movement, weight support and coordination following recovery after SCI [29]. One week prior to SCI, all animals were trained and evaluated to exclude animals with any intrinsic motor dysfunction. After injury, the BBB open field test was performed by 2 examiners blinded to the experimental procedures to assess the spontaneous locomotor recovery on days 1, 3, 7, 14, 21 and 28.

In the first experimental block, 21 animals were used for total mRNA extraction at specific times post-SCI. The open-field test was carried out to establish a homogeneous group, and only animals with a BBB score of 0–3 one day after surgery and with similar functional progress were included in the study.

In the second experimental block, 62 animals were used to evaluate the effects of IP leptin administration. The BBB scale and the BBB sub-score were used to analyse the differences in functional recovery due to leptin administration. In this regard, the BBB scores were further analysed by calculating sub-scores [58]–[60], which allows for characterisation of the individual aspects of locomotion, alone or in combination.

To assess the effects of leptin treatment on locomotor gait dynamics, we used the CatWalk® gait analysis system (version 7.1; Noldus) as previously described [61],[62]. Individual footprints were visualised by slightly dampening the rats' feet with a 50% glycerol solution. Animals subjected to SCI and IP administration of vehicle or leptin were trained for 5 min daily in the week preceding functional assessment. For each animal, a minimum of 5 uninterrupted runs, at a consistent pace, were used either before (to obtain pre-lesioned basal reference values) or at the completion of the study. To simplify the study and allow for a quick and easy interpretation of the functional outcome, we chose 3 well-established parameters for foot prints analysis [63]: the BOS, print position, and angle parameters.

#### 2. Evaluation of sensory recovery

Sensory function was assessed by analysing thermal hyperalgesia and mechanical allodynia at the end of the study (28 d post-SCI) in animals subjected to SCI and IP administration.

-Thermal hyperalgesia: the thermal sensitivity of the hind paw plantar surface was tested by means of a Hargreaves Plantar Test Apparatus (Ugo Basile), as previously described [64]. Paw withdrawal latency in response to a heating stimulus was performed in 3 consecutive trials on each animal, with a cutoff latency of 20 s to avoid tissue damage, at both 3 days before injury to establish pre-lesioned basal reference values and 28 d after injury. A decrease in withdrawal latency of >3 s was considered as neuropathic pain.

-Mechanical allodynia: at the same time points when thermal hyperalgesia was assessed, the tactile sensory response to mechanical stimuli was evaluated by stimulation of the hind paw plantar surface with a calibrated set of von Frey filaments (Stoelting Co. Wood Dale, IL). Briefly, animals were placed in individual Plexiglas chambers with a plastic mesh floor for at least 30 min. Filaments were then applied to the bending point for 6 s, and brisk paw withdrawal was considered as a positive response. The response threshold was determined as the lowest force that provoked a minimum of 50% positive retractions from a total of 20 stimulations per paw [65], [66].

The animals from the different experimental groups were randomly tested by two observers blinded to the treatments.

### Electrophysiological assessment

An electrophysiological assessment of spinal cord function was performed by brain stimulation and hind limb MEPs recording in both limbs of each animal at the end of the study (28 d post-SCI), as previously described with minor modifications [67]. Briefly, animals were anesthetised with pentobarbital (40 mg/kg i.p.) and placed in a prone position on a homoeothermic blanket to maintain their body temperature at 37°C. First, the lumbar circuitry state was assessed by means of electrical stimulation of the sciatic nerve with single electrical pulses of 0.1 milliseconds (ms) duration and supra-maximal intensity delivered by monopolar needles percutaneously inserted at the sciatic notch. The compound muscle potential (cMAP) of the gastrocnemius and tibialis anterior muscles was recorded by means of needle electrodes, with the active electrode on the belly of the muscle and the reference at the second toe with Signal software (Cambridge Electronic Design Ltd.). The latency and amplitude of the average direct muscle response (M-wave) resulting from 5 consecutive recordings was analysed using the same software. Without moving the recording electrodes, stimulation electrodes were placed subcutaneously over the skull, with the anode over the motor cortex and the cathode on the hard palate, to deliver pulses of 2 ms duration and supramaximal intensity. A total of 20–25 independent recordings of MEPs were acquired, amplified and filtered (10–1000 Hz) from the gastrocnemius and tibialis anterior muscles of both limbs of each animal. The resulting average recordings were analysed for latency, amplitude and rectified area (Modulus) of the negative peak. Values are represented as the group mean and SEM of the average values resulting from the gastrocnemius and tibialis anterior muscles of both limbs of each animal. To ensure recording confidence, 2 additional animals were used in a preliminary experiment to corroborate a total lack of elicited MEPs after a whole spinal cord transection (data not shown).

### mRNA isolation and RT-qPCR

Multiple mRNA transcripts were quantified by RT-qPCR including leptin, ObRb, PPARs, IL-1β, IL-6, TNF-α, IRF-1, NF-_k_β, catalase, GPx, iNOS and decorin.

Total RNA was isolated from a 1-cm-long spinal cord fragment that contained the wound epicentre using the RNeasy Lipid Mini Kit (Qiagen). Reverse transcription from 3 µg of DNase-treated RNA and RT-qPCR was carried out using specific primers (Table 1), as previously described [57].

**Table 1:** RT-qPCR primers.

Gene name	Forward primer	Reverse primer	Acc no.
Catalase	5′-GTACAGGCCGGCTCTCACA-3′	5′-ACCCGTGCTTTACAGGTTAGCT-3′	NM_012520
Decorin	5′-AGCAACCCTGTCCGGTATTG-3′	5′-CGCCCGAAGACACATCTGA-3′	NM_024129
GPx	5′-GCTGTGCGCGCTCCAT-3′	5′-ACCATGTGCCCATCGATGT-3′	NM_017165
IL-1β	5′-GACCTGTTCTTTGAGGCTGACA-3′	5′-AGTCAAGGGCTTGGAAGCAA-3′	NM_031512
IL-6	5′-CCCACCAGGAACGAAAGTCA-3′	5′-GCGGAGAGAAACTTCATAGCTGTT-3′	NM_012589
iNOS	5′-TGGTGAAAGCGGTGTTCTTTG-3′	5′-ACGCGGGAAGCCATGA-3′	NM_012611
IRF-1	5-AACTTCCGGTGTGCCATGA-3′	5′-TCCTGCTCTGGTCCTTCACTTC-3′	NM_012591
Leptin	5′-CTTCATTCCCGGGCTTCA-3′	5′-GCCAGGGTCTGGTCCATCTT-3′	NM_013076
NF-_k_β	5′-TCGTGAGGGATCTGCTAGAAGTG-3′	5′-GTTGCCTCCAGATCCACAAAC-3′	NM_001107095
PPARα	5-AGGCCTCAGGATACCACTATGG-3′	5-CCGAAAGAAGCCCTTGCA-3′	NM_013196
PPARβ/δ	5-GCTCACTGACAGATGAGGACAAA-3′	5-CTGGGTCTGAGCGCAGATG-3′	NM_013141
PPARγ	5-CCCACCAACTTCGGAATCAG-3′	5-GGAATGGGAGTGGTCATCCA-3′	NM_013124
TNF-α	5′-CCCAGAAAAGCAAGCAACCA-3′	5′-GCCTCGGGCCAGTGTATG-3′	NM_012675

The primers used for RT-qPCR analysis of the genes assessed in this study, including the gene symbol, primer sequences (forward and reverse sequences, respectively) and the Genbank accession number.

### Western blot

Denatured protein samples (100 µg) were resolved on 10% SDS-PAGE gels, transferred to PVDF membranes and incubated with a primary antibody raised against p-CREB (1∶500; Santa Cruz Technology), Bcl-x_L_ (1∶500; Abcam), Bcl-2 (1∶250; Abcam), p-STAT3 Tyr705 (1∶1000; Cell Signaling Technology) or Cox-2 (1∶500; BD Laboratories). A corresponding anti-rabbit or anti-mouse horseradish peroxidase (HRP)-conjugated secondary antibody (1∶7000; Amersham) was used as previously described [57].

### Caspase-3 activity

Caspase-3 activity was measured using the Caspase-Glo Assay System (Promega) according to the manufacturer's instructions. Briefly, 50 µl of the Caspase-Glo buffer was added to 100 µg of total protein in a 96-well plate. After incubating at RT for 60 min, the luminescence was measured on an Infinite M200 microplate reader (Tecan). Standard curves were used to determine the linearity of the responses.

### Histology and IHC

Animals were anesthetised with pentobarbital and perfused intracardially with 150 ml of heparinised saline solution followed by 1 ml/g of 4% paraformaldehyde. Spinal cord fragments 2 cm in length (1 cm rostral and 1 cm caudal to the wound epicentre) were immediately dissected, post-fixed for 4 h in the same fixative solution, and then cryoprotected by immersion in 30% sucrose for 48 h and embedded in Neg-50 medium (Richard-Allan Scientific) and stored at −20°C. Parallel serial cryosections (30 µm thick) were then cut, mounted on slides and stored at −20°C.

Cellular ObRb expression was evaluated in different cell types in a set of parallel sections from uninjured (n = 5) and from vehicle and leptin-treated animals sacrificed at 24 h post-injury (see table S1) by dual IHC against adenomatous polyposis coli (APC; oligodendrocytes), NeuN (neurons), GFAP (astrocytes) or OX-42 (microglia/macrophages). Briefly, sections were treated for 1 h at RT with a Blocking Buffer (BB) containing 10% FBS, 0.3% BSA and 0.3% Triton X-100 in (pH 7.4) TBS, followed by incubation overnight at 4°C plus 1 h at RT with a rabbit anti-ObRb (1∶100; Abcam) primary antibody in BB. After washing, the sections were incubated for 1 h at RT with an Alexa 488-linked anti-rabbit secondary antibody (1∶1000; Invitrogen) in BB with 10% rat serum instead of FBS. The sections were then washed again and blocked with BB for 1 h at RT before immunostaining as above but with an Alexa 594-linked anti-mouse antibody and one of the following primary mouse antibodies: APC (1∶100; Calbiochem), NeuN (1∶250; Chemicon), GFAP (1∶1000; Sigma) or OX-42 (1∶500; Wako). Finally, the slides were coverslipped in “Immumount” (Thermo Scientific) and confocal images were collected at RT on a Leica TCS SP5 confocal microscope, with 40×1.25 NA and 63×1.40 NA oil objectives and LAS-AF software (Leica).

To evaluate astroglial and microglia/macrophage reactivity, GFAP and Iba1 IHC were performed on a set of parallel sections from each animal euthanised at the end of the study. Briefly, sections were treated for 10 min with 2% H_2_O_2_ in a 70% methanol solution at RT, immersed for 1 h at RT with BB and incubated overnight at 4°C plus 1 h at RT with rabbit anti-GFAP (1∶1000; Dako) or rabbit anti-Iba1 (1∶1000; Wako). After washing, the sections were incubated with a biotinylated anti-rabbit secondary antibody (1∶500; Vector) in BB and then with HRP-linked streptavidin (1∶500; Perkin Elmer) and visualised using the Nova Red Kit (Vector), according to the manufacturer's instructions. The sections were dehydrated in graded ethanol and coverslipped in DPX (Panreac). Images were acquired on a BX61 Motorized Research Microscope (Olympus) and the immunoreactive volume and area were quantified with ImageJ software, either in the total spinal cord volume or at each rostro-caudal spinal cord level analysed (8 sections per animal separated by 600 µm and containing the wound epicentre). The data corresponding to the total immunoreactive volume and area per rostro-caudal level are presented, respectively, as the percentage of total spinal cord volume and spinal cord area per rostro-caudal level.

In each single IHC experiment, processed sections lacking primary antibody were used as controls. For dual immunohistochemistry and to identify putative cross reactivity between the second secondary antibody and the first primary antibody, processed sections lacking the second primary antibody were used as controls.

Myelin preservation was assessed by eriochrome cyanine staining (ECy) in 1 set of parallel sections from each animal at 28 d post-SCI. Briefly, slides were air-dried for 2 h, dehydrated in acetone for 5 min and stained by immersion in 0.2% eriochrome cyanine plus 0.4% iron alum for 30 min. Gray matter (gray) and white (dark blue) matter were differentiated by incubation in 5% iron alum, followed by 1% Borax and 1.25% potassium ferricyanide for 15 min each. Finally, sections were rinsed with tap water, dehydrated with graded ethanol solutions, cleared with xylol and coverslipped with DPX. Myelin preserved areas were analysed by densitometry with ImageJ software. The data corresponding to preserved myelin are presented as the percentage of the spinal cord area in each rostro-caudal level.

### Statistical analysis

All values are expressed as the mean ± SEM. In the analysis of locomotion in an open field, groups were compared using two-way repeated measures ANOVA, followed by a Bonferroni post-hoc analysis. The nonparametric Mann-Whitney U test was used to determine differences in the withdrawal latency and threshold force in the response to heating or von Frey filament stimulation. Data from the CatWalk® gait analysis, electrophysiological assessment, IHC, western blot and RT-qPCR were compared using a one-way ANOVA followed by a Tukey post-hoc and t-tests for a point-to-point comparison. In all cases, P<0.05 was considered to be statistically significant. All statistical analyses were performed using GraphPad Prism (version 4.0).

## Supporting Information

Figure S1 **The principal categories within the BBB scale are improved by leptin treatment.** (A) Significantly more rats in the leptin treatment group recovered the ability to step by 7 d post-SCI when compared with vehicle treated rats. (B) There was a significantly improved toe clearance over time in leptin-treated rats at 14, 21 and 28 d post-SCI. (C) Paw position subscores, due to more frequent parallel paw placements, increased over time in the leptin-treated animals when compared with the vehicle controls. (D) Finally, the leptin-treated group displayed more frequent forelimb-hindlimb coordination than the vehicle-treated rats at 21 and 28 d post-SCI. In all cases, values represent the mean ± SEM; *p<0.05 versus the vehicle control group.
(TIF)

Table S1 **The distribution of animals subjected to SCI and IP leptin/vehicle administration (experiment I).**
(DOC)

Table S2 **The distribution of animals subjected to SCI and IP leptin/vehicle administration (experiment II).**
(DOC)
