# Supplementary figures and images for: Acute Leptin Treatment Enhances Functional Recovery after Spinal Cord Injury
Source: PLoS One. 2012 Apr 20;7(4):e35594. doi: 10.1371/journal.pone.0035594 (PMC3334982; doi:10.1371/journal.pone.0035594)

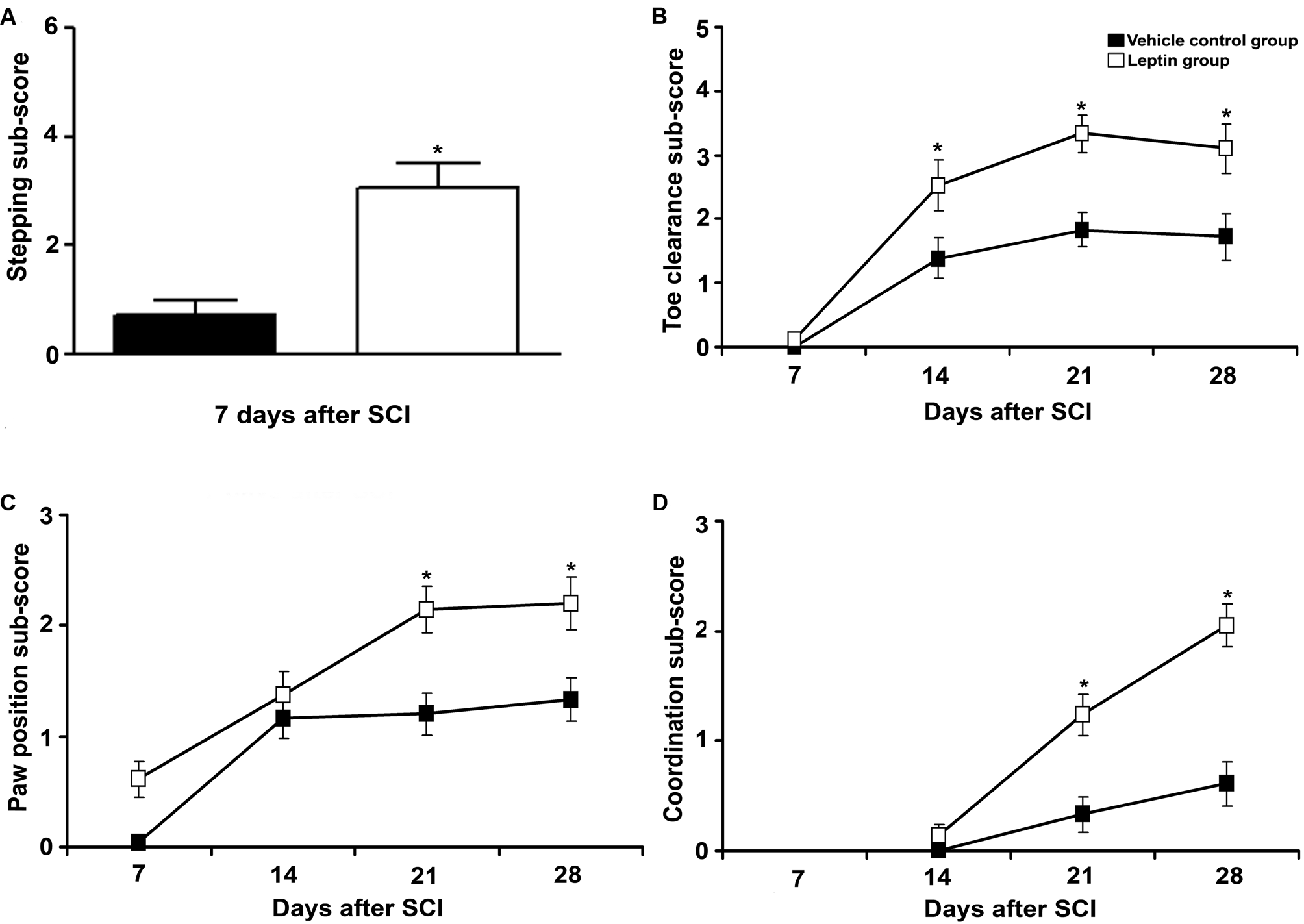

Supplement: Figure S1 — The principal categories within the BBB scale are improved by leptin treatment. (A) Significantly more rats in the leptin treatment group recovered the ability to step by 7 d post-SCI when compared with vehicle treated rats. (B) There was a significantly improved toe clearance over time in leptin-treated rats at 14, 21 and 28 d post-SCI. (C) Paw position subscores, due to more frequent parallel paw placements, increased over time in the leptin-treated animals when compared with the vehicle controls. (D) Finally, the leptin-treated group displayed more frequent forelimb-hindlimb coordination than the vehicle-treated rats at 21 and 28 d post-SCI. In all cases, values represent the mean ± SEM; *p<0.05 versus the vehicle control group. (TIF) [file pone.0035594.s001.tif]
