# Supplementary material for: Acute Leptin Treatment Enhances Functional Recovery after Spinal Cord Injury
Source: PLoS One. 2012 Apr 20;7(4):e35594. doi: 10.1371/journal.pone.0035594 (PMC3334982; doi:10.1371/journal.pone.0035594)
Supplement: Table S1 — The distribution of animals subjected to SCI and IP leptin/vehicle administration (experiment I). (DOC) [file pone.0035594.s002.doc]

**Table S1.** The distribution of animals subjected to SCI and IP leptin/vehicle administration (experiment I).

|  |  |  | **Times post-SCI** |  |
| --- | --- | --- | --- | --- |
| **GROUP I** | **TECHNIQUE** | **24 h** | **7 d** | **28 d** |
| **CONTROL** | **RT-qPCR/Western Blot** | 3 | 3 | 3 |
|  | **Histology/IHC** | 3 | - | 3 |
|  | **CatWalk gait analysis** | - | - | 5* |
|  | **Sensory function** | - | - | 5* |
|  | **Electrophysiolog**y | - | - | 5* |
| **LEPTIN** | **RT-qPCR/Western Blot** | 3 | 3 | 3 |
|  | **Histology/IHC** | 3 | - | 4 |
|  | **CatWalk gait analysis** | - | - | 7* |
|  | **Sensory function** | - | - | 7* |
|  | **Electrophysiology** | - | - | 7* |

* The functional assessment was performed in the same animals used for RT-qPCR and Histology/IHC at 28 d post-SCI.
