# Supplementary material for: Acute Leptin Treatment Enhances Functional Recovery after Spinal Cord Injury
Source: PLoS One. 2012 Apr 20;7(4):e35594. doi: 10.1371/journal.pone.0035594 (PMC3334982; doi:10.1371/journal.pone.0035594)
Supplement: Table S2 — The distribution of animals subjected to SCI and IP leptin/vehicle administration (experiment II). (DOC) [file pone.0035594.s003.doc]

**Table S2.** The distribution of animals subjected to SCI and IP leptin/vehicle administration (experiment II).

|  |  | **Times post-SCI** | | |
| --- | --- | --- | --- | --- |
| **GROUP II** | **TECHNIQUE** | **24 h** | **7 d** | **28 d** |
| **CONTROL** | **Histology/IHC** | - | - | 15(8)** |
|  | **CatWalk gait analysis** | - | - | 7* |
|  | **Sensory function** | - | - | 7* |
|  | **Electrophysiology** | - | - | 15* |
| **LEPTIN** | **Histology/IHC** | - | - | 16(8)** |
|  | **CatWalk gait analysis** | - | - | 8* |
|  | **Sensory function** | - | - | 8* |
|  | **Electrophysiology** | - | - | 16* |

* The functional assessment was performed in the same animals used for Histology/IHC at 28 d post-SCI.

** The number of animals used for GFAP and Iba1 Histology/IHC.
